# Waist circumference as a vital sign in clinical practice: a Consensus Statement from the IAS and ICCR Working Group on Visceral Obesity

**DOI:** 10.1038/s41574-019-0310-7

**Published:** 2020-02-04

**Authors:** Robert Ross, Ian J. Neeland, Shizuya Yamashita, Iris Shai, Jaap Seidell, Paolo Magni, Raul D. Santos, Benoit Arsenault, Ada Cuevas, Frank B. Hu, Bruce A. Griffin, Alberto Zambon, Philip Barter, Jean-Charles Fruchart, Robert H. Eckel, Yuji Matsuzawa, Jean-Pierre Després

**Affiliations:** 1 School of Kinesiology and Health Studies, School of Medicine, Department of Endocrinology and Metabolism, Queen’s University, Kingston, ON Canada; 2 Department of Internal Medicine, Division of Cardiology, University of Texas Southwestern Medical Center, Dallas, TX USA; 3 Departments of Cardiovascular Medicine and Community Medicine, Osaka University Graduate School of Medicine, Osaka, Japan; 4 Faculty of Health Sciences, Ben-Gurion University of the Negev, Beer-Sheva, Israel; 5 Department of Health Sciences and the EMGO Institute for Health and Care Research, VU University Amsterdam, Amsterdam, Netherlands; 6 Department of Pharmacological and Biomolecular Sciences, Universita’ degli Studi di Milano, Milan, Italy; 7 Scientific Institute for Research, Hospitalization and Health Care (IRCCS) MultiMedica, Sesto San Giovanni, Italy; 8 Lipid Clinic Heart Institute (InCor), University of São Paulo, Medical School Hospital, São Paulo, Brazil; 9 Hospital Israelita Albert Einstein, Sao Paulo, Brazil; 10 Department of Kinesiology, Faculty of Medicine, Université Laval, Québec, QC Canada; 11 Department of Clinical Nutrition and Metabolism, Clínica Las Condes, Santiago, Chile; 12 Departments of Nutrition and Epidemiology, Harvard T.H. Chan School of Public Health, Boston, MA USA; 13 Department of Nutritional Sciences, University of Surrey, Guildford, UK; 14 Department of Medicine - DIMED, University of Padua, Padova, Italy; 15 School of Medical Sciences, University of New South Wales Australia, Sydney, NSW Australia; 16 Fondation Cœur et Artères, Lille, France; 17 Division of Endocrinology, Metabolism and Diabetes, and Division of Cardiology, Anschutz University of Colorado School of Medicine, Aurora, CO USA; 18 Department of Endocrinology and Metabolism, Sumitomo Hospital, Osaka, Japan; 19 Department of Medicine, Faculty of Medicine, Université Laval, Québec, QC Canada

**Keywords:** Disease prevention, Obesity, Metabolic syndrome, Predictive markers

## Abstract

Despite decades of unequivocal evidence that waist circumference provides both independent and additive information to BMI for predicting morbidity and risk of death, this measurement is not routinely obtained in clinical practice. This Consensus Statement proposes that measurements of waist circumference afford practitioners with an important opportunity to improve the management and health of patients. We argue that BMI alone is not sufficient to properly assess or manage the cardiometabolic risk associated with increased adiposity in adults and provide a thorough review of the evidence that will empower health practitioners and professional societies to routinely include waist circumference in the evaluation and management of patients with overweight or obesity. We recommend that decreases in waist circumference are a critically important treatment target for reducing adverse health risks for both men and women. Moreover, we describe evidence that clinically relevant reductions in waist circumference can be achieved by routine, moderate-intensity exercise and/or dietary interventions. We identify gaps in the knowledge, including the refinement of waist circumference threshold values for a given BMI category, to optimize obesity risk stratification across age, sex and ethnicity. We recommend that health professionals are trained to properly perform this simple measurement and consider it as an important ‘vital sign’ in clinical practice.

## Abstract (web summary)

In this Consensus Statement, the International Atherosclerosis Society and International Chair on Cardiometabolic Risk Working Group on Visceral Obesity recommend that waist circumference be included routinely as a measurement in clinical practice. They summarize the evidence that waist circumference and BMI together can provide improved assessments of cardiometabolic risk compared with either measurement alone.

## Introduction

The prevalence of adult overweight and obesity as defined using BMI has increased worldwide since the 1980s, with no country demonstrating any successful declines in the 33 years of recorded data^1^. Obesity is a major public health problem worldwide^2^ and reliance on measurements of BMI alone has proven inadequate to help clinicians assess and manage obesity-related health risk in their patients. For instance, although many individuals with overweight or obesity will develop cardiometabolic health complications such as type 2 diabetes mellitus (T2DM) and cardiovascular disease (CVD) during their lifetimes, a sizeable minority will remain free of these chronic diseases, a phenomenon that has been described as metabolically healthy obesity (MHO).

The prevalence of MHO among adults varies greatly between studies owing to differences in age, ethnicity and environmental factors, as well as the lack of a universal definition of metabolic health and a universal classification system for obesity^3^. Furthermore, studies with long-term follow-up periods have generally found that MHO is often a temporary or transition state for most individuals with obesity. For example, in a study with a 20-year follow-up, approximately half of adults with MHO (defined in this study as having less than two cardiometabolic parameters that fall outside of healthy ranges) became metabolically unhealthy by the end of the study. Moreover, study participants with MHO were at increased risk of cardiovascular events after long-term follow-up^4^. Similarly, a study considering the full range of possible definitions for MHO suggested that the risk of a cardiovascular event associated with the MHO phenotype increased with longer follow-up times. Furthermore, similar CVD risk estimates were observed when MHO was defined by criteria other than the absence of the metabolic syndrome^5^. Despite the fact that the limitations of BMI as an index for obesity have been known for decades, several obesity guidelines worldwide remain steadfast in the recommendation that BMI alone be the measure to characterize obesity-related morbidity and risk of death^6–9^.

The failure of BMI to fully capture cardiometabolic risk is partially related to the fact that BMI in isolation is an insufficient biomarker of abdominal adiposity. Waist circumference is a simple method to assess abdominal adiposity that is easy to standardize and clinically apply. Waist circumference is strongly associated with all-cause^10,11^ and cardiovascular mortality^12,13^ with or without adjustment for BMI^10,14^. However, the full strength of the association between waist circumference with morbidity and mortality is realized only after adjustment for BMI^10,15,16^. Thus, waist circumference enables a further refinement of the adverse health risk characterized by BMI and this measurement should be included when stratifying obesity-related health risk. Indeed, resistance to the routine inclusion of waist circumference in clinical practice not only ignores the evidence of its utility, but fails to take advantage of opportunities to counsel patients regarding the higher-risk phenotype of obesity. In addition, the measurement of both BMI and waist circumference will provide unique opportunities to follow the utility of treatment and effectiveness of interventions designed to manage obesity and related metabolic disease.

In 2017, the International Atherosclerosis Society (IAS) and International Chair on Cardiometabolic Risk (ICCR) Working Group on Visceral Obesity convened in Prague, Czech Republic, to discuss the importance of abdominal obesity as a risk factor for premature atherosclerosis and CVD in adults (Supplementary Information). The group agreed to work on the development of consensus documents which would reflect the position of the two organizations. In this Consensus Statement, we summarize the evidence that BMI alone is not sufficient to properly assess, evaluate or manage the cardiometabolic risk associated with increased adiposity and recommend that waist circumference be adopted as a routine measurement in clinical practice alongside BMI to classify obesity.

## Methodology

This Consensus Statement is designed to provide the consensus of the IAS and ICCR Working Group (Supplementary Information) on waist circumference as an anthropometric measure that improves patient management. The Consensus Statement was developed as follows. The first face-to-face meeting occurred on 24 April 2017 to review the high-quality evidence available and known to the subject experts. After discussion and deliberation amongst the experts regarding the context and quality of the evidence, an executive writing group (R.R., I.J.N., J.-P.D., J.S. and Y.M.) was appointed and tasked with writing the first draft. The draft was subsequently circulated to all authors for critical revision of intellectual content pertinent to each authors’ expertise. High-quality published literature that became available after the initial face-to-face meeting (through June 2019) was identified by all authors and reviewed by the executive writing group for inclusion in the manuscript. The first author coordinated the final preparation and submission of the Consensus Statement after the group achieved consensus and approved its content.

## Historical perspective

The importance of body fat distribution as a risk factor for several diseases (for example, CVD, hypertension, stroke and T2DM) and mortality has been recognized for several decades. In 1956, Jean Vague was the first to show the importance of fat distribution in relation to various diseases, describing what he termed ‘android’ and ‘gynoid’ types of obesity^17^. These classifications were later interpreted by Ahmed Kissebah and colleagues as upper versus lower body fat accumulation as reflected by a high or low waist–hip circumference ratio (WHR), respectively^18^. The upper and lower body fat accumulation phenotypes were based on body morphology as assessed by external anthropometric measures such as skinfolds and circumferences.

The WHR increased in popularity when epidemiologists in the USA and Sweden showed that WHR, separately or in combination with BMI, was associated with increased risk of death, CVD and T2DM^19–22^, findings that were subsequently confirmed in many studies. However, later evidence indicated that, compared with the WHR, waist circumference alone was more strongly associated with the absolute amount of intra-abdominal or visceral fat, the fat depot that conveys the strongest health risk^23,24^. Furthermore, when a ratio such as WHR is used to follow changes in regional adipose depots, the utility of the ratio is limited when both the numerator and denominator values change in response to treatment. Consequently, the combination of WHR and BMI for assessing obesity risk were replaced by single threshold values for waist circumference alone^25^. The NIH was the first to use the threshold values for waist circumference (≥88 cm in women and ≥102 cm in men) as suggested by Michael Lean and colleagues, in combination with a classification of overall obesity as assessed by BMI^25^. Although the use of these specific waist circumference values to identify white adults with abdominal obesity remains a cornerstone of obesity guidelines worldwide, we present evidence to challenge the supportive rationale and provide evidence in support of alternative waist circumference values to be used in concert with BMI.

As an alternative to measurements of waist circumference, the WHR or waist–thigh circumference ratio, Margaret Ashwell and others proposed the waist–height ratio as a measure of abdominal obesity^26,27^. Compared with the previous measurements, the waist–height ratio shows similar and sometimes slightly stronger associations with the risk of CVD or T2DM^28,29^. An explanation for why adding height increases the prediction of disease risk might be because short stature is associated with increased risk of CVD^30^. In growing children and adolescents, the waist–height ratio could be more useful for the classification of abdominal obesity than waist circumference alone. However, in fully grown adults, the waist–height ratio is less useful as height is generally fixed and the value can only be altered by changes in waist circumference. Moreover, height is only marginally associated with waist circumference^31^. For the assessment of the effectiveness of lifestyle changes in adults, waist circumference might be preferred as a simple tool. Other alternatives to waist circumference have included the conicity index^32^ and the abdominal obesity index^33^, but they are, at best, only slightly better predictors of disease risk than waist circumference alone.

## Prevalence of abdominal obesity

Despite a strong association between waist circumference and BMI at the population level, emerging evidence suggests that, across populations, waist circumference might be increasing beyond what is expected according to BMI. In other words, the phenotype of obesity might be changing over time to one that reflects an increase in abdominal adiposity^34^. For example, Ian Janssen and colleagues examined the changes in waist circumference for a given BMI over a 30-year period in a Canadian sample^35^. Notably, for a given BMI, Canadians had a larger waist circumference in 2007 compared with 1981. Specifically, the researchers observed a waist circumference that was greater by 1.1 cm in men and 4.9 cm in women for a BMI of 25 kg/m^2^ between 1981 and 2007. Similarly, Sandra Albrecht and colleagues examined the secular changes in waist circumference in the USA (1988–2007), England (1992–2008), China (1993–2011) and Mexico (1999–2012)^36^ and reported statistically significantly increased waist circumference values relative to BMI in all countries studied and in most subpopulations.

**Fig. 1:**
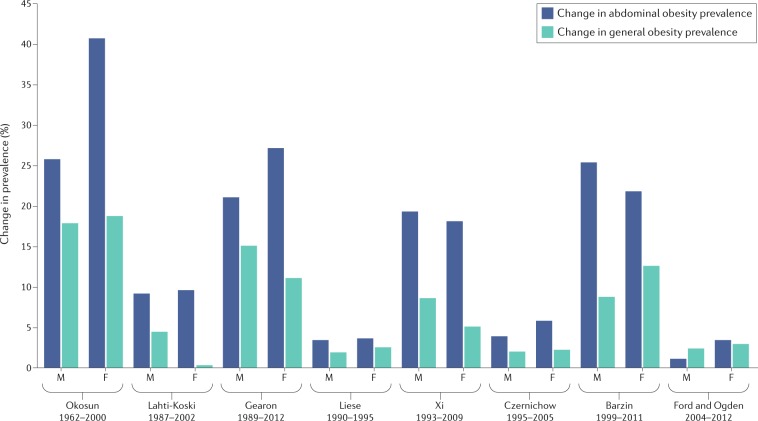
The prevalence of abdominal obesity and obesity measured in different studies. Changes in the prevalence of abdominal obesity (measured using waist circumference) and general obesity (measured using BMI) measured in different studies during the time period indicated on the *x* axis. General obesity was defined as BMI ≥30 kg/m^2^. Abdominal obesity was defined as waist circumference ≥88 cm and ≥102 cm for women and men, respectively. However, Xi et al. defined general obesity as BMI ≥28 kg/m^2^ and abdominal obesity as waist circumference ≥80 cm and ≥85 cm for Chinese women and men, respectively^113^. In addition, Barzin et al. defined general obesity as BMI ≥30 kg/m^2^ and abdominal obesity as waist circumference ≥91 cm and ≥89 cm for Iranian women and men, respectively^114^. Years given (for example, 1999–2011) indicate the years in which data were collected. F, female; M, male. Data are from refs^37,113–121^.

These observations are consistent with those of Tommy Visscher and colleagues, who performed an extensive review and concluded that the majority of the evidence suggests a trend in which the relative increases in waist circumference were larger than the relative increases in BMI^37^. This observation is seemingly independent of age, sex and ethnicity, as few groups failed to demonstrate the general trend of secular waist circumference increasing beyond that expected by BMI (Fig. 1). The failure of BMI to detect such an increase in abdominal obesity confirms the limitations of BMI alone to identify the phenotype of obesity that conveys the greatest health risk.

### Conclusions and recommendations — prevalence of abdominal obesity

- Although the prevalence of obesity measured by BMI might have plateaued in some countries, the prevalence of abdominal obesity as measured by waist circumference is generally increasing.
- The lack of inclusion of waist circumference in global obesity surveillance might inadequately characterize the health risk associated with the global obesity prevalence, as it seems that the prevalence of abdominal obesity is increasing.
- Current obesity prevalence trends based on BMI alone should be interpreted with caution. We recommend that serious consideration should be given to the inclusion of waist circumference in obesity surveillance studies.

## Identifying the high-risk obesity phenotype

### Waist circumference, BMI and health outcomes — categorical analysis

It is not surprising that waist circumference and BMI alone are positively associated with morbidity^15^ and mortality^13^ independent of age, sex and ethnicity, given the strong association between these anthropometric variables across cohorts. However, it is also well established that, for any given BMI, the variation in waist circumference is considerable, and, in any given BMI category, adults with higher waist circumference values are at increased adverse health risk compared with those with a lower waist circumference^38–40^. This observation is well illustrated by James Cerhan and colleagues, who pooled data from 11 prospective cohort studies with 650,386 white adults from the USA, Australia and Sweden aged 20–83 years^11^. In this study, the authors observed that waist circumference was positively associated with mortality within every BMI category examined, from 20 kg/m^2^ to 50 kg/m^2^. This finding is consistent with that of Ellen de Hollander and colleagues, who performed a meta-analysis involving over 58,000 predominantly white older adults from around the world and reported that the age-adjusted and smoking-adjusted mortality was substantially greater for those with an elevated waist circumference within normal weight, overweight and obese categories as defined by BMI^41^. The ability of waist circumference to add to the adverse health risk observed within a given BMI category provides the basis for the current classification system used to characterize obesity-related health risk^8,42^.

### Waist circumference, BMI and health outcomes — continuous analysis

Despite the observation that the association between waist circumference and adverse health risk varies across BMI categories^11^, current obesity-risk classification systems recommend using the same waist circumference threshold values for all BMI categories^42^. We propose that important information about BMI and waist circumference is lost when they are converted from continuous to broad categorical variables and that this loss of information affects the manner in which BMI and waist circumference predict morbidity and mortality. Specifically, when BMI and waist circumference are considered as categorical variables in the same risk prediction model, they are both positively related to morbidity and mortality^38^. However, when BMI and waist circumference are considered as continuous variables in the same risk prediction model, risk prediction by waist circumference improves, whereas the association between BMI and adverse health risk is weakened^10,43^. The full strength of the association between waist circumference with morbidity and/or mortality is not fully realized until adjustment for BMI^11,12,41^.

Evidence in support of adjusting waist circumference for BMI comes from Janne Bigaard and colleagues who report that a strong association exists between waist circumference and all-cause mortality after adjustment for BMI^43^. For example, a 10% larger waist circumference corresponded to a 1.48 (95% CI 1.36–1.61) times higher mortality over the whole range of waist circumference in both men and women after adjustment for BMI. This observation was confirmed by Tobias Pischon and colleagues, who observed that the highest quintile of waist circumference (≥102.7 cm in men and ≥89.0 cm in women) was associated with an increased risk of all-cause death of 1.33 (95% CI 1.24–1.44) before BMI adjustment, with an increased risk of death of 2.05 (95% CI 1.80–2.33) after adjustment for BMI^10^.

Consistent with observations based on asymptomatic adults, Thais Coutinho and colleagues report similar observations for a cohort of 14,284 adults with CVD who were followed up for 2.3 years (5,696 deaths). The cohort was divided into tertiles for both waist circumference and BMI. In comparison with the lowest waist circumference tertile, a significant association with risk of death was observed for the highest tertile for waist circumference after adjustment for age, sex, smoking, diabetes mellitus, hypertension and BMI (HR 1.29, 95% CI 1.20–1.39). By contrast, after adjustment for age, sex, smoking, diabetes mellitus, hypertension and waist circumference, increasing tertiles of BMI were inversely associated with risk of death (HR 0.64, 95% CI 0.59–0.69)^44^.

The findings from this systematic review^44^ are partially confirmed by Diewertje Sluik and colleagues, who examined the relationships between waist circumference, BMI and survival in 5,435 individuals with T2DM over 4.6 years of follow-up (interquartile range 2.0–9.8 years)^45^. In this prospective cohort study, the cohort was divided into quintiles for both BMI and waist circumference. After adjustment for T2DM duration, insulin treatment, prevalent myocardial infarction, stroke, cancer, smoking status, smoking duration, educational level, physical activity, alcohol consumption and BMI, the HR for risk of death associated with the highest tertile was 2.11 (95% CI 1.23–3.61) in comparison with the lowest waist circumference quintile. By contrast, in comparison with the lowest quintile for BMI (adjusted for the same variables, with waist circumference replacing BMI), the HR for risk of death for the highest BMI quintile was 0.33 (95% CI 0.19–0.60). In summary, when associations between waist circumference and BMI with morbidity and mortality are considered in continuous models, for a given waist circumference, the higher the BMI the lower the adverse health risk.

Why the association between waist circumference and adverse health risk is increased following adjustment for BMI is not established. It is possible that the health protective effect of a larger BMI for a given waist circumference is explained by an increased accumulation of subcutaneous adipose tissue in the lower body^46^. For example, in a study of >2,000 older participants from the Health, Ageing and Body Composition study, Marieke Snijder and colleagues were among the first to report that thigh adipose tissue mass is negatively associated with glucose intolerance and dyslipidaemia, after accounting for abdominal adipose tissue mass^47^. This observation was confirmed by Sophie Eastwood and colleagues, who reported that in South Asian adults the protective effects of total subcutaneous adipose tissue for T2DM and HbA_1c_ levels emerge only after accounting for visceral adipose tissue (VAT) accumulation^48^.

**Fig. 2:**
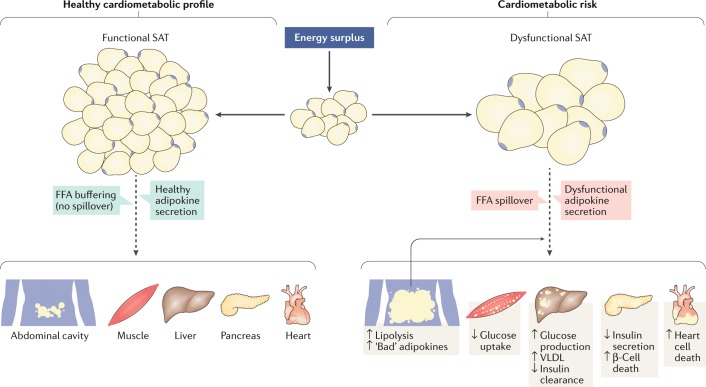
Overview of potential role of functional and dysfunctional adipose tissue contributing to increased cardiometabolic risk. The ability of subcutaneous adipose tissue (SAT) to expand through hyperplasia (generation of new fat cells) allows the safe storage of the excess energy from the diet into a properly expanding subcutaneous ‘metabolic sink’. When this process becomes saturated or in situations where adipose tissue has a limited ability to expand, there is a spillover of the excess energy, which must be stored in visceral adipose tissue as well as in normally lean organs such as the skeletal muscle, the liver, the pancreas and the heart, a process described as ectopic fat deposition. Visceral adiposity is associated with a hyperlipolytic state resistant to the effect of insulin along with an altered secretion of adipokines including inflammatory cytokines whereas a set of metabolic dysfunctions are specifically associated with increased skeletal muscle, liver, pancreas, and epicardial, pericardial and intra-myocardial fat. FFA, free fatty acid.

A causal mechanism has not been established that explains the attenuation in morbidity and mortality associated with increased lower body adiposity for a given level of abdominal obesity. We suggest that the increased capacity to store excess energy consumption in the gluteal–femoral subcutaneous adipocytes might protect against excess lipid deposition in VAT and ectopic depots such as the liver, the heart and the skeletal muscle (Fig. 2). Thus, for a given waist circumference, a larger BMI might represent a phenotype with elevations in lower body subcutaneous adipose tissue. Alternatively, adults with elevations in BMI for a given waist circumference could have decreased amounts of VAT. Excess lipid accumulation in VAT and ectopic depots is associated with increased cardiometabolic risk^47–49^. Moreover, VAT is an established marker of morbidity^50,51^ and mortality^24,52^. These findings provide a plausible mechanism by which lower values for BMI or hip circumference for a given waist circumference would increase adverse health risk.

This notion is reinforced by Jennifer Kuk and colleagues who reported that BMI is an independent and positive correlate of VAT in adults before adjustment for waist circumference; however, BMI is negatively associated with VAT mass after adjustment for waist circumference^53^. This study also reported that, after adjustment for waist circumference, BMI was positively associated with lower body subcutaneous adipose tissue mass and skeletal muscle mass. These observations support the putative mechanism described above and, consequently, that the negative association commonly observed between BMI and morbidity and mortality after adjustment for waist circumference might be explained by a decreased deposition of lower body subcutaneous adipose tissue and muscle mass, an increased accumulation of visceral adiposity, or both.

In summary, the combination of BMI and waist circumference can identify the highest-risk phenotype of obesity far better than either measure alone. Although guidelines for the management of obesity from several professional societies recognize the importance of measuring waist circumference, in the context of risk stratification for future cardiometabolic morbidity and mortality, these guidelines limit the recommendation to measure waist circumference to adults defined by BMI to have overweight or obesity. On the basis of the observations described in this section, waist circumference could be just as important, if not more informative, in persons with lower BMI, where an elevated waist circumference is more likely to signify visceral adiposity and increased cardiometabolic risk. This observation is particularly true for older adults^54^.

### Conclusions and recommendations — identifying the high-risk obesity phenotype

- In categorical analyses, waist circumference is associated with health outcomes within all BMI categories independent of sex and age.
- When BMI and waist circumference are considered as continuous variables in the same risk prediction model, waist circumference remains a positive predictor of risk of death, but BMI is unrelated or negatively related to this risk.
- The strength of the association between waist circumference and morbidity and/or mortality is not fully realized until after adjustment for BMI.
- The improved ability of waist circumference to predict health outcomes over BMI might be at least partially explained by the ability of waist circumference to identify adults with increased VAT mass.
- We recommend that measurements of waist circumference and BMI should become a standard part of clinical encounters (that is, an accepted ‘vital sign’).

## Importance in clinical settings

For practitioners, the decision to include a novel measure in clinical practice is driven in large part by two important, yet very different questions. The first centres on whether the measure or biomarker improves risk prediction in a specific population for a specific disease. For example, does the addition of a new risk factor improve the prognostic performance of an established risk prediction algorithm, such as the Pooled Cohort Equations (PCE) or Framingham Risk Score (FRS) in adults at risk of CVD? The second question is concerned with whether improvement in the new risk marker would lead to a corresponding reduction in risk of, for example, cardiovascular events. In many situations, even if a biomarker does not add to risk prediction, it can still serve as an excellent target for risk reduction. Here we consider the importance of waist circumference in clinical settings by addressing these two questions.

### Risk prediction

The evaluation of the utility of any biomarker, such as waist circumference, for risk prediction requires a thorough understanding of the epidemiological context in which the risk assessment is evaluated. In addition, several statistical benchmarks need to be met in order for the biomarker to improve risk prediction beyond traditional measures. These criteria are especially important for waist circumference, as established sex-specific and ethnicity-specific differences exist in waist circumference threshold levels^55,56^. In 2009, the American Heart Association published a scientific statement on the required criteria for the evaluation of novel risk markers of CVD^57^, followed by recommendations for assessment of cardiovascular risk in asymptomatic adults in 2010 (ref.^58^). Novel biomarkers must at the very least have an independent statistical association with health risk, after accounting for established risk markers in the context of a multivariable epidemiological model. This characteristic alone is insufficient, however, as many novel biomarkers meet this minimum standard yet do not meaningfully improve risk prediction beyond traditional markers. More stringent benchmarks have therefore been developed to assess biomarker utility, which include calibration, discrimination^58^ and net reclassification improvement^59^. Therefore, to critically evaluate waist circumference as a novel biomarker for use in risk prediction algorithms, these stringent criteria need to be applied.

Numerous studies demonstrate a statistical association between waist circumference and mortality and morbidity in epidemiological cohorts. For example, a systematic review and meta-regression analysis of 18 studies comprising >680,000 European participants with up to 24 years of follow-up demonstrated that waist circumference was associated with increased all-cause death above values of 95 cm for men and 80 cm for women^60^. Notably, increased waist circumference above these thresholds was associated with increased relative risk of all-cause death, even among those with normal BMI (20.0–24.9 kg/m^2^)^60^. In the USA, prospective follow-up over 9 years of 14,699 black, white and mixed ethnicity participants in the Atherosclerosis Risk in Communities study showed that waist circumference was associated with increased risk of coronary heart disease (553 events; RR 1.37, 95% CI 1.21–1.56) but not with all-cause death^61^.

Despite the existence of a robust statistical association with all-cause death independent of BMI, there is no solid evidence that addition of waist circumference to standard cardiovascular risk models (such as FRS^62^ or PCE^63^) improves risk prediction using more stringent statistical benchmarks. For example, a study evaluating the utility of the PCE across WHO-defined classes of obesity^42^ in five large epidemiological cohorts comprised of ~25,000 individuals assessed whether risk discrimination of the PCE would be improved by including the obesity-specific measures BMI and waist circumference^64^. The researchers found that although each measure was individually associated (BMI: HR 1.04, 95% CI 1.02–1.07; waist circumference: HR 1.11, 95% CI 1.09–1.13 per 1 SD increase) with increased risk of atherosclerotic CVD, no significant improvement occurred in the c-statistic with the addition of either BMI or waist circumference to the other PCE variables^64^. Similarly, a pooled analysis of four French population studies including >20,000 participants assessed the utility of additional risk factors when added to the FRS for 10-year coronary heart disease risk prediction^65^. The researchers found that BMI (*P* = 0.03) but not waist circumference (*P* = 0.42) remained associated with risk of coronary heart disease when added to FRS, but the addition of either factor did not improve model discrimination^65^.

On the basis of these observations alone, one might conclude that the measure of waist circumference in clinical settings is not supported as risk prediction is not improved. However, Nancy Cook and others have demonstrated how difficult it is for the addition of any biomarker to substantially improve prognostic performance^59,66–68^. Indeed, Michael Pencina and colleagues estimated that the nonmodifiable risk factors of age, sex and ethnicity capture 63–80% of the prognostic performance of cardiovascular risk models, and that adding systolic blood pressure, plasma levels of non-HDL cholesterol, diabetes mellitus or smoking to a model with other risk factors increases prognostic performance as measured by the c-statistic by only 0.004–0.013 (ref.^69^). Furthermore, any additive value of waist circumference to risk prediction algorithms could be overwhelmed by more proximate, downstream causative risk factors such as elevated blood pressure and abnormal plasma concentrations of glucose. In other words, waist circumference might not improve prognostic performance as, independent of BMI, waist circumference is a principal driver of alterations in downstream cardiometabolic risk factors. A detailed discussion of the merits of different approaches (for example, c-statistic, net reclassification index and discrimination index) to determine the utility of novel biomarkers to improve risk prediction is beyond the scope of this article and the reader is encouraged to review recent critiques to gain insight on this important issue^66,69^.

### Risk reduction

Whether the addition of waist circumference improves the prognostic performance of established risk algorithms is a clinically relevant question that remains to be answered; however, the effect of targeting waist circumference on morbidity and mortality is an entirely different issue of equal or greater clinical relevance. Several examples exist in the literature where a risk marker might improve risk prediction but modifying the marker clinically does not impact risk reduction. For example, a low level of HDL cholesterol is a central risk factor associated with the risk of coronary artery disease in multiple risk prediction algorithms, yet raising plasma levels of HDL cholesterol pharmacologically has not improved CVD outcomes^70^. Conversely, a risk factor might not meaningfully improve statistical risk prediction but can be an important modifiable target for risk reduction. Indeed, we argue that, at any BMI value, waist circumference is a major driver of the deterioration in cardiometabolic risk markers or factors and, consequently, that reducing waist circumference is a critical step towards reducing cardiometabolic disease risk.

**Fig. 3:**
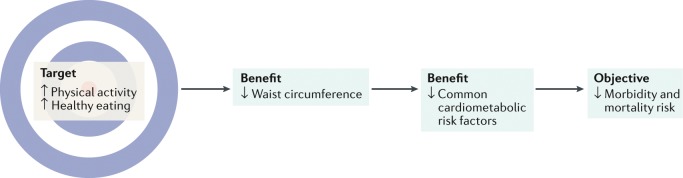
Waist circumference is a modifiable risk factor that can indicate cardiometabolic risk, morbidity and mortality. An illustration of the important role that decreases in waist circumference have for linking improvements in lifestyle behaviours with downstream reductions in the risk of morbidity and mortality. The benefits associated with reductions in waist circumference might be observed with or without a change in BMI.

As we described earlier, waist circumference is well established as an independent predictor of morbidity and mortality, and the full strength of waist circumference is realized after controlling for BMI. We suggest that the association between waist circumference and hard clinical end points is explained in large measure by the association between changes in waist circumference and corresponding cardiometabolic risk factors. For example, evidence from randomized controlled trials (RCTs) has consistently revealed that, independent of sex and age, lifestyle-induced reductions in waist circumference are associated with improvements in cardiometabolic risk factors with or without corresponding weight loss^71–76^. These observations remain consistent regardless of whether the reduction in waist circumference is induced by energy restriction (that is, caloric restriction)^73,75,77^ or an increase in energy expenditure (that is, exercise)^71,73–75^. We have previously argued that the conduit between change in waist circumference and cardiometabolic risk is visceral adiposity, which is a strong marker of cardiometabolic risk^24^. Taken together, these observations highlight the critical role of waist circumference reduction through lifestyle behaviours in downstream reduction in morbidity and mortality (Fig. 3).

In summary, whether waist circumference adds to the prognostic performance of cardiovascular risk models awaits definitive evidence. However, waist circumference is now clearly established as a key driver of altered levels of cardiometabolic risk factors and markers. Consequently, reducing waist circumference is a critical step in cardiometabolic risk reduction, as it offers a pragmatic and simple target for managing patient risk.

### Conclusions and recommendations — waist circumference and risk prediction

- The combination of BMI and waist circumference identifies a high-risk obesity phenotype better than either measure alone.
- We recommend that waist circumference should be measured in clinical practice as it is a key driver of risk; for example, many patients have altered CVD risk factors because they have abdominal obesity.
- Waist circumference is a critical factor that can be used to measure the reduction in CVD risk after the adoption of healthy behaviours.

## A highly responsive vital sign

Evidence from several reviews and meta-analyses confirm that, regardless of age and sex, a decrease in energy intake through diet or an increase in energy expenditure through exercise is associated with a substantial reduction in waist circumference^78–87^. For studies wherein the negative energy balance is induced by diet alone, evidence from RCTs suggest that waist circumference is reduced independent of diet composition and duration of treatment^88^. Whether a dose–response relationship exists between a negative energy balance induced by diet and waist circumference is unclear.

Although it is intuitive to suggest that increased amounts of exercise would be positively associated with corresponding reductions in waist circumference, to date this notion is not supported by evidence from RCTs^71,74,89–91^. For example, Robert Ross and colleagues conducted a large RCT whereby participants (*n* = 300) were assigned to either a control arm or an intervention arm with different exercise levels: low, defined as 180 kcal per session for women and 300 kcal per session for men; and high, defined as 360 kcal per session for women and 600 kcal per session for men^74^. A doubling of the energy expenditure induced by exercise did not result in a difference in waist circumference reduction between the exercise groups. However, all intervention groups significantly reduced waist circumference (~5 cm) compared with the control arm (*P* < 0.001)^74^. These findings are consistent with the findings of Christopher Slentz and colleagues, who reported no difference in waist circumference or VAT reduction between low-level (14 kcal/kg body weight per week, *n* = 46) and high-level (23 kcal/kg body weight per week, *n* = 42) exercise groups^90,91^. In addition, Timothy Church and colleagues conducted an RCT whereby participants were prescribed different levels of exercise (low, 4 kcal/kg body weight per week, *n* = 155; moderate, 8 kcal/kg body weight per week, *n* = 104; or high, 12 kcal/kg body weight per week, *n* = 103) that was matched for intensity (50% peak oxygen consumption (VO_2_peak))^71^. A significant reduction was observed in waist circumference across all exercise groups compared with the no-exercise controls, with no difference between the different prescribed levels^71^.

Few RCTs have examined the effects of exercise intensity on waist circumference^74,90–92^. A small trial conducted by Brian Irving and colleagues observed that a high-intensity exercise (over the lactate threshold 3 days per week and under the lactate threshold 2 days per week) group (*n* = 9) had significantly reduced waist circumference compared with a low-intensity (under the lactate threshold 5 days per week) group (*n* = 11)^92^. However, no significant differences were observed in VAT reduction by single slice CT between high-intensity and low-intensity groups. A large RCT conducted by Slentz and colleagues observed that an increase in exercise intensity from moderate (40–55% VO_2_peak, *n* = 40) to vigorous (65–80% VO_2_peak, *n* = 42) intensity was not associated with differences in waist circumference reduction^90,91^. However, the researchers did not fix the level of exercise between the intensity groups, which might explain their observations. Ross and colleagues controlled the amount of energy expenditure between moderate-intensity (50% VO_2_peak, *n* = 76) and high-intensity (75% VO_2_peak, *n* = 76) exercise groups^74^. Their observations are consistent with those of Slentz and colleagues, whereby differences in exercise intensity did not affect waist circumference reductions. These findings are consistent with a meta-analysis carried out in 2017 wherein no difference in waist circumference reduction was observed between high-intensity interval training and moderate-intensity exercise^93^. In summary, current evidence suggests that increasing the intensity of exercise interventions is not associated with a further decrease in waist circumference.

VAT mass is not routinely measured in clinical settings, so it is of interest whether reductions in waist circumference are associated with corresponding reductions in VAT. Although evidence from systematic reviews and meta-analyses demonstrate an association between reductions in waist circumference and VAT^79,82,84,85,94^, the shared variance is modest (~40%)^75,95,96^. Of note, to our knowledge every study that has reported a reduction in waist circumference has also reported a corresponding reduction in VAT. Thus, although it is reasonable to suggest that a reduction in waist circumference is associated with a reduction in VAT mass, a precise estimation of individual VAT reduction from waist circumference is not possible. Nonetheless, the corresponding reduction of VAT with waist circumference in a dose-dependent manner highlights the importance of routine measurement of waist circumference in clinical practice. Of particular interest to practitioners, several reviews have observed significant VAT reduction in response to exercise in the absence of weight loss^80,85^.

### Conclusions and recommendations — changes in waist circumference in response to treatment

- Exercise and/or diet consistent with guideline recommendations are associated with substantial reductions in waist circumference, independent of age, sex or ethnicity.
- Available evidence from RCTs suggests that exercise is associated with substantial reductions in waist circumference, independent of the quantity or intensity of exercise.
- Exercise-induced or diet-induced reductions in waist circumference are observed with or without weight loss.
- We recommend that practitioners routinely measure waist circumference as it provides them with a simple anthropometric measure to determine the efficacy of lifestyle-based strategies designed to reduce abdominal obesity.

## Measurement of waist circumference

The emergence of waist circumference as a strong independent marker of morbidity and mortality is striking given that there is no consensus regarding the optimal protocol for measurement of waist circumference. Moreover, the waist circumference protocols recommended by leading health authorities have no scientific rationale. In 2008, a panel of experts performed a systematic review of 120 studies to determine whether measurement protocol influenced the relationship between waist circumference, morbidity and mortality, and observed similar patterns of association between the outcomes and all waist circumference protocols across sample size, sex, age and ethnicity^97^. Upon careful review of the various protocols described within the literature, the panel recommended that the waist circumference protocol described by the WHO guidelines^98^ (the midpoint between the lower border of the rib cage and the iliac crest) and the NIH guidelines^99^ (the superior border of the iliac crest) are probably more reliable and feasible measures for both the practitioner and the general public. This conclusion was made as both waist circumference measurement protocols use bony landmarks to identify the proper waist circumference measurement location.

The expert panel recognized that differences might exist in absolute waist circumference measures due to the difference in protocols between the WHO and NIH methods. However, few studies have compared measures at the sites recommended by the WHO and NIH. Jack Wang and colleagues reported no difference between the iliac crest and midpoint protocols for men and an absolute difference of 1.8 cm for women^100^. These observations were confirmed by Caitlin Mason and Peter Katzmarzyk, who reported no difference between the iliac crest (NIH) and midpoint (WHO) protocols for men and an absolute difference of ~2 cm for women^101^. More importantly, Mason and Katzmarzyk reported that the prevalence estimates of abdominal obesity (here defined as waist circumference >88 cm for women and >102 cm for men) identified using the iliac crest and midpoint protocols were about 32% for both protocols in men and 47% and 41% for the iliac crest and midpoint protocols, respectively, in women^101^. Consequently, although adopting a standard approach to waist circumference measurement would add to the utility of waist circumference measures for obesity-related risk stratification, the prevalence estimates of abdominal obesity in predominantly white populations using the iliac crest or midpoint protocols do not seem to be materially different.

Of note, the observation that the NIH and WHO protocols do not substantially differ is not consistent with those made by Yumi Matsushita and colleagues, who sampled 940 Japanese adults and reported that the mean difference between the iliac crest and midpoint protocols for men was ~2 cm, whereas for women the difference was ~9 cm (refs^102,103^). However, the waist circumference measurements assessed at the two sites had a similar ability to screen for the metabolic syndrome, as defined by National Cholesterol Education Program, in a cohort of 1,140 Japanese adults^102^.

Several investigations have evaluated the relationship between self-measured and technician-measured waist circumference^104–108^. Instructions for self-measurement of waist circumference are often provided in point form through simple surveys^108^. Good agreement between self-measured and technician-measured waist circumference is observed, with strong correlation coefficients ranging between 0.8 and 0.9 for both men and women. However, both men and women tend to underestimate their waist circumference measures compared with the technician-measured values, with differences ranging between about 1 cm and 3 cm. Moreover, high BMI and large baseline waist circumference are associated with a larger degree of under-reporting^105,107^. Overall these observations are encouraging and suggest that self-measures of waist circumference can be obtained in a straightforward manner and are in good agreement with technician-measured values.

Instructional videos that provide a detailed illustration of the step-by-step procedures for both technician-measurement and self-measurement of waist circumference can be freely accessed at myhealthywaist (http://www.myhealthywaist.org/evaluating-cmr/clinical-tools/waist-circumference-measurement-guidelines/index.html).

### Conclusions and recommendations — measurement of waist circumference

- Currently, no consensus exists on the optimal protocol for measurement of waist circumference and little scientific rationale is provided for any of the waist circumference protocols recommended by leading health authorities.
- The waist circumference measurement protocol has no substantial influence on the association between waist circumference, all-cause mortality and CVD-related mortality, CVD and T2DM.
- Absolute differences in waist circumference obtained by the two most often used protocols, iliac crest (NIH) and midpoint between the last rib and iliac crest (WHO), are generally small for adult men but are much larger for women.
- The classification of abdominal obesity might differ depending on the waist circumference protocol.
- We recommend that waist circumference measurements are obtained at the level of the iliac crest or the midpoint between the last rib and iliac crest. The protocol selected to measure waist circumference should be used consistently.
- Self-measures of waist circumference can be obtained in a straightforward manner and are in good agreement with technician-measured values.

## Threshold values to estimate risk

Current guidelines for identifying obesity indicate that adverse health risk increases when moving from normal weight to obese BMI categories. Moreover, within each BMI category, individuals with high waist circumference values are at increased risk of adverse health outcomes compared with those with normal waist circumference values^109^. For example, a single waist circumference threshold for white adults (men >102 cm; women >88 cm) is currently used to denote a high waist circumference, regardless of BMI category. Of note, these sex-specific thresholds were originally developed using cross-sectional data in white adults, among whom a waist circumference of 102 cm in men and 88 cm in women corresponded to a BMI of 30.0 kg/m^2^, which is the BMI threshold for obesity^109^. Thus, these waist circumference threshold values were designed to be used in place of BMI as an alternative way to identify obesity and consequently were not developed based on the relationship between waist circumference and adverse health risk.

In order to address this limitation, Christopher Ardern and colleagues developed and cross-validated waist circumference thresholds within BMI categories in relation to estimated risk of future CVD (using FRS)^110^. The utility of the derived values was compared with the single waist circumference thresholds (women >88 cm; men >102 cm) recommended by leading health authorities. The results of their study revealed that the current recommendations that use a single waist circumference threshold across all BMI categories are insufficient to identify those at increased health risk. In both sexes, the use of BMI category-specific waist circumference thresholds improved the identification of individuals at a high risk of future coronary events, leading the authors to propose BMI-specific waist circumference values (Table 1). In 2009, Harpreet Bajaj and colleagues compared the prognostic performance of the Ardern waist circumference values (Table 1) with the traditional waist circumference values (men >102 cm; women >88 cm) for all-cause mortality in a large cohort of 5,453 predominantly white adults with high cardiometabolic risk^111^. For both men and women, the Ardern waist circumference values substantially improved predictions of mortality compared with the traditional values. These observations are promising and support, at least for white adults, the clinical utility of the BMI category-specific waist circumference thresholds given in Table 1.

**Table 1:** Waist circumference thresholds

BMI category (kg/m^2^)	Waist circumference (cm)^a^	
	Women	Men
Normal weight (18.5–24.9)	≥80	≥90
Overweight (25–29.9)	≥90	≥100
Obese I (30–34.9 )	≥105	≥110
Obese II and III (≥35 )	≥115	≥125

Table provides waist circumference thresholds stratified by BMI for white individuals; individuals with measurements higher than these values have a high risk of future coronary events (based on 10-year risk of coronary events or the presence of diabetes mellitus). ^a^Waist circumference threshold indicating increased health risk within each BMI category. Data were originally presented in ref.^110^.

Of note, BMI-specific waist circumference thresholds have been developed in African American and white men and women^112^. Similar to previous research, the optimal waist circumference thresholds increased across BMI categories in both ethnic groups and were higher in men than in women. However, no evidence of differences in waist circumference occurred between ethnicities within each sex^112^.

Pischon and colleagues investigated the associations between BMI, waist circumference and risk of death among 359,387 adults from nine countries in the European Prospective Investigation into Cancer and Nutrition cohort^10^. In this study, the authors confirmed that, for a given BMI in men and women, the risk of death increased by 17% in men and 13% in women for every 5 cm increase in waist circumference. Although the waist circumference values that optimized prediction of the risk of death for any given BMI value were not reported, the findings reinforce the notion that waist circumference thresholds increase across BMI categories and that the combination of waist circumference and BMI provide improved predictions of health risk than either anthropometric measure alone.

Ethnicity-specific values for waist circumference that have been optimized for the identification of adults with elevated CVD risk have been developed (Table 2). With few exceptions, the values presented in Table 2 were derived using cross-sectional data and were not considered in association with BMI. The range in high-risk waist circumference values for both adult men (80–98 cm) and women (80–96 cm) varies considerably across ethnicities, which confirms the need for ethnicity-specific waist circumference values. Prospective studies using representative populations are required to firmly establish ethnicity-specific and BMI category-specific waist circumference threshold values that distinguish adults at increased health risk.

**Table 2:** Ethnicity-specific thresholds

Ethnic group	Waist circumference (cm)^a^	Ref.
***Japanese***^b^		
Men	≥85	^122^
Women	≥90	^122^
***Jordanian***		
Men	≥98	^123^
Women	≥96	^123^
***Chinese***		
Men	≥80	^124^
Women	≥80	^124^
***Korean***		
Men	≥90	^125^
Women	≥85	^125^
***Tunisian***		
Men	≥85	^126^
Women	≥85	^126^
***Iranian***		
Men	≥89	^127^
Women	≥91	^127^
***Asian Indian***		
Men	≥90	^128^
Women	≥80	^128^

^a^Waist circumference values for adults above which cardiometabolic risk is elevated. ^b^Japanese waist circumference values are thresholds above which visceral adipose tissue volume is >100 cm^3^.

As noted above, the ethnicity-specific waist circumference values in Table 2 were optimized for the identification of adults with elevated CVD risk. The Japanese waist circumference values, however, were optimized for identification of men and women with CT-measured VAT values >100 cm^3^ at the level of the umbilicus (navel)^112^. The rationale for using VAT as the outcome was that cardiometabolic risk was found to increase substantially at this VAT level for adult Japanese men and women^56^. Accordingly, Japanese threshold values for waist circumference were established at 85 cm in men and 90 cm in women, which corresponded to the VAT threshold of 100 cm^3^ (ref.^112^).

### Conclusions and recommendations — values of waist circumference to estimate health risk

- From the evidence available, we question the rationale behind current guidelines recommending that a single waist circumference threshold for white adults (men >102 cm; women >88 cm) be used to denote a high waist circumference, regardless of BMI category.
- We recommend that prospective studies using representative populations are carried out to address the need for BMI category-specific waist circumference thresholds across different ethnicities (such as those proposed in Table 1 for white adults). This recommendation does not, however, diminish the importance of measuring waist circumference to follow changes over time and, hence, the utility of strategies designed to reduce abdominal obesity and associated health risk.

## Conclusions

The main recommendation of this Consensus Statement is that waist circumference should be routinely measured in clinical practice, as it can provide additional information for guiding patient management. Indeed, decades of research have produced unequivocal evidence that waist circumference provides both independent and additive information to BMI for morbidity and mortality prediction. On the basis of these observations, not including waist circumference measurement in routine clinical practice fails to provide an optimal approach for stratifying patients according to risk. The measurement of waist circumference in clinical settings is both important and feasible. Self-measurement of waist circumference is easily obtained and in good agreement with technician-measured waist circumference. Numerous epidemiological studies and RCTs have now demonstrated that reductions in waist circumference can be achieved by routine, moderate-intensity exercise and/or diet changes.

Gaps in our knowledge still remain, and refinement of waist circumference threshold values for a given BMI category across different ages, by sex and by ethnicity will require further investigation. To address this need, we recommend that prospective studies be carried out in the relevant populations. Despite these gaps in our knowledge, overwhelming evidence presented here suggests that the measurement of waist circumference improves patient management and that its omission from routine clinical practice for the majority of patients is no longer acceptable. Accordingly, the inclusion of waist circumference measurement in routine practice affords practitioners with an important opportunity to improve the care and health of patients. Health professionals should be trained to properly perform this simple measurement and should consider it as an important vital sign to assess and identify, as an important treatment target in clinical practice.

## Supplementary information

Supplementary Information

## Glossary

Calibration: The ability to correctly predict the proportion of participants in a given group who will experience an event.
Discrimination: The probability of a diagnostic test or risk prediction instrument to distinguish between higher and lower risk.
Net reclassification improvement: The relative increase in the predicted probabilities for individuals who experience events and the decrease for individuals who do not.
C-statistic: A measure of goodness-of-fit for binary outcomes in a logistic regression model.
VO_2_peak: The highest value of VO_2_ (that is, oxygen consumption) attained during an incremental or other high-intensity exercise test.
Lactate threshold: The exercise intensity at which the blood concentration of lactate and/or lactic acid begins to exponentially increase.
Iliac crest: The superior border of the wing of the ilium.
