# Supplementary material for: Waist circumference as a vital sign in clinical practice: a Consensus Statement from the IAS and ICCR Working Group on Visceral Obesity
Source: Nat Rev Endocrinol. 2020 Feb 4;16(3):177–89. doi: 10.1038/s41574-019-0310-7 (PMC7027970; doi:10.1038/s41574-019-0310-7)
Supplement: Supplementary file 1 — Supplementary Information [file 41574_2019_310_MOESM1_ESM.pdf]

nature REVIEWS ENDOCRINOLOGY

SUPPLEMENTARY INFORMATION
In format as provided by the authors

Waist circumference as a vital sign in clinical practice: a Consensus Statement from the IAS and ICCR Working Group on Visceral Obesity

Robert Ross, Ian J. Neeland, Shizuya Yamashita, Iris Shai, Jaap Seidell, Paolo Magni, Raul D. Santos, Benoit Arsenault, Ada Cuevas, Frank B. Hu, Bruce A. Griffin, Alberto Zambon, Philip Barter, Jean-Charles Fruchart, Robert H. Eckel, Yuji Matsuzawa and Jean-Pierre Després

https://doi.org/10.1038/s41574-019-0310-7

Supplementary Table 1: ICCR-IAS Visceral Adiposity Working Group

ICCR-IAS Visceral Adiposity Working Group

Benoit Arsenault
Québec Heart and Lung Institute
2725, chemin Sainte-Foy, Y-2110
Québec QC G1V 4G5
CANADA
E-mail: benoit.arsenault@criucpq.ulaval.ca

Philip Barter
School of Medical Sciences
University of New South Wales
Sydney, NSW, 2052,
AUSTRALIA
E-mail: pbarter@ozemail.com.au
p.barter@unsw.edu.au

Ada Cuevas
Chair, Dept. Clinical Nutrition Department
Clinica Las Condes
Lo Fontecilla 441 Las Condes
Santiago,
CHILE
E-mail: acuevas@clinicalascondes.cl

Jean-Pierre Després
Professor, Department of Kinesiology
Faculty of Medicine, Université Laval
Director of Research in Cardiology
Québec Heart and Lung Institute
Director of Science and Innovation
Alliance santé Québec
Scientific Director
International Chair on Cardiometabolic Risk
2725, chemin Sainte-Foy, A-2087
Québec QC G1V 4G5
CANADA
E-mail: jean-pierre.despres@criucpq.ulaval.ca

Robert H. Eckel
Professor of Medicine
Division of Endocrinology, Metabolism and Diabetes
School of Medicine
University of Colorado
12801 E. 17th Avenue, Room 7107 8106
Aurora, CO 80045
USA
E-mail: robert.eckel@ucdenver.edu

Jean-Charles Fruchart
Professor Institut Pasteur de Lille

Professor Emeritus University Lille II
Fondation “Coeur et Artères”
256, Avenue Eugène Avinée
F-59120 Loos
FRANCE
E-mail: jeancharles.fruchart@yahoo.fr

Bruce A. Griffin
Professor of Nutritional Metabolism
Department of Nutritional Sciences
University of Surrey
Guildford, Surrey
United Kingdom GU2 7WG
E-mail: b.griffin@surrey.ac.uk

Frank B. Hu
Professor of Nutrition and Epidemiology
Harvard T.H. Chan School of Public Health
Professor of Medicine
Harvard Medical School and
Brigham and Women’s Hospital
BOSTON, MA
USA
E-mail: frank.hu@channing.harvard.edu

Paolo Magni
Laboratory of Clinical di Pathology
Dept Pharmacologica and Biomolecular Sciences (DiSFeB)
University of Milan
via Balzaretti, 9
20133 Milano
ITALY
E-mail: paolo.magni@unimi.it

Yuji Matsuzawa
Director
Sumitomo Hospital, 5-3-20, Nakanoshima
Kita-Ku
530-0005 Osaka
JAPAN
E-mail: matsuzawa-yuji@sumitomo-hp.or.jp

Ian J. Neeland
Assistant Professor of Medicine
Dedman Family Scholar in Clinical Care
Division of Cardiology, Department of Internal Medicine
UT Southwestern Medical Center
5323 Harry Hines Blvd.
Dallas, TX 75390-8830
USA
E-mail: ian.neeland@utsouthwestern.edu

Robert Ross
Professor, School of Kinesiology and Health Studies
School of Medicine, Department of Endocrinology and Metabolism
Kinesiology Building, 28 Division Street, Room 301E

Queen's University
Kingston, ON K7L 3N6
CANADA
E-mail: rossr@queensu.ca

Raul D. Santos
Lipid Clinic Heart Institute (InCor)
University of São Paulo, Medical School Hospital
and Hospital Israelita Albert Einstein
Avenue Dr. Eneas C. Aguiar 44, Bloco 2
Sao Paulo, Segundo Andar, Sala 4, CEP-05403-900
BRAZIL
E-mail: rdsf@uol.com.br

Jacob C. Seidell
Professor of Nutrition and Health
Department of Health Sciences
VU University of Amsterdam
De Boelelaan 1085
1081 HV Amsterdam
THE NETHERLANDS
E-mail: j.c.seidell@vu.nl

Iris Shai
Professor of Nutrition and Epidemiology
Adviser to BGU President on the Advancement of
of Women in Academia
Dep. of Public Health, Faculty of Health Sciences
Be'er Sheva
ISRAEL
E-mail: irish@bgu.ac.il

Shizuya Yamashita
Director, Rinku General Medical Center
2-23 Ourai-kita, Rinku, Izumisano, Osaka 598-8577, Japan
Professor, Department of Community Medicine & Department of Cardiovascular Medicine
Osaka University Graduate School of Medicine
2-2 Yamadaoka, Suita, Osaka 565-0871
JAPAN
E-mail: shizu@imed2.med.osaka-u.ac.jp

Alberto Zambon
Associate Professor of Medicine
Department of Medicine – DIMED
Medical School
University of Padua
Via Giustiniani, 2
35128 Padova
ITALY
E-mail: alberto.zambon@unipd.it
